# Alcohol, Hormones, and Postmenopausal Women

**Published:** 1998

**Authors:** Matthew P. Longnecker, Marilyn Tseng

**Affiliations:** Matthew P. Longnecker, M.D., Sc.D., is a medical research officer at the Epidemiology Branch, National Institute of Environmental Health Sciences, Research Triangle Park, North Carolina. Marilyn Tseng, Ph.D. is an assistant member at the Division of Population Science, Fox Chase Cancer Center, Philadelphia, Pennsylvania

**Keywords:** AODE (alcohol and other drug effects), menopause, female, sex hormones, estrogens, mammary gland, neoplastic disease, cardiovascular disorder, moderate AOD use, drug therapy, literature review

## Abstract

Many women take supplemental estrogens after menopause, a practice called hormone replacement therapy (HRT). Moderate alcohol consumption may increase estrogen levels in women receiving HRT, potentially affecting their risk for various adverse health effects. Two recent studies, however, provide no strong evidence for an effect of alcohol on hormones in postmenopausal women. The possible association between alcohol consumption and risk of cancer of the breast does not appear to be mediated by estrogens. Both estrogens and moderate alcohol consumption have been associated with a decreased risk for cardiovascular disease; however, alcohol’s beneficial effect on heart disease does not appear to involve hormonal mechanisms. Additional research is needed to define the consequences of moderate drinking on hormone levels after menopause.

Approximately 30 percent of American women are older than age 50, the average age of menopause (Cramer and Xu 1996).^1^ Epidemiological studies suggest that approximately 50 percent of women in this age group consume at least moderate quantities of alcohol (National Institute on Alcohol Abuse and Alcoholism [NIAAA] 1997).^2^ Therefore, any adverse effects of alcohol among this population could have a significant effect on public health.

Menopause is characterized by greatly diminished levels^3^ of a group of steroid reproductive hormones called estrogens. Estrogens (e.g., estrone and estradiol) travel through the bloodstream and exert widespread physiological effects on organ growth and development. Approximately 25 percent of postmenopausal women take supplemental estrogens to alleviate unpleasant symptoms of menopause, a practice called hormone replacement therapy (HRT) (Ginsburg et al. 1996). Estrogens have various effects on the health of postmenopausal women (Rich-Edwards and Hennekens 1996). For example, HRT decreases the risk of cardiovascular disease and osteoporosis^4^ but may increase the risk of breast cancer (Ginsburg et al. 1996). Recent experimental studies show that moderate alcohol consumption may increase estrogen levels in post-menopausal women receiving HRT (Ginsburg et al. 1996).

This article reviews some of the basic hormonal changes associated with menopause, examines the effects of alcohol consumption on estrogen levels after menopause, and discusses the relationship of postmenopausal alcohol use to the risks of cardiovascular disease and cancer. Although several reproductive hormones may play important roles in the menopausal transition, this article focuses on estrogens, in part because the health effects of altered estrogen levels are relatively well understood. For a discussion of alcohol’s effects on post-menopausal women with alcoholic cirrhosis, see Gavaler (1995).

## Hormonal Function Before Menopause

The female reproductive cycle is governed primarily by two groups of hormones: the gonadotropins and the steroid reproductive hormones. The gonadotropins include follicle-stimulating hormone (FSH) and luteinizing hormone (LH), which are produced by the pituitary gland and released into the bloodstream. The steroid reproductive hormones are produced largely in the ovaries. Approximately every 28 days, levels of FSH and LH in the blood begin to increase, stimulating the growth and development of specialized cell clusters (i.e., follicles) within the ovaries. Each follicle nurtures a developing egg cell (i.e., ovum) and secretes estrogens. Within the ovaries, estrogens help promote follicular development. A midcycle surge of LH secretion from the pituitary gland causes the release of a mature ovum, which then migrates away from the ovaries. Under the influence of LH, the ovary continues to secrete estrogens and other steroid hormones to prepare the body for pregnancy and lactation. If the ovum is not fertilized, gonadotropin and ovarian hormone levels decline until the next cycle is about to begin.

These cyclic events are orchestrated by complex interactions among many hormones and other physiological substances. For example, the ovaries release hormones called activin and inhibin, which, respectively, stimulate and suppress gonadotropin production. In addition, the pituitary gland adjusts the magnitude and timing of gonadotropin secretion in response to levels of ovarian hormones in the blood. This mechanism, called feedback regulation, helps fine tune hormone levels and promotes the orderly progression of physiological events. Hormonal balance also is influenced by metabolic reactions that can synthesize specific hormones from other hormones to which they are structurally related.

## Hormonal Changes During Menopause

The number of functional ovarian follicles decreases with age. This decrease accelerates after approximately age 40 (Burger 1996*a*); thus, by the time a woman experiences her final menstrual period (i.e., menopause), the ovaries contain virtually no follicles (Burger 1996*b*). The postmenopausal state is characterized by unvarying hormone levels and the absence of ovulatory cycling.

The approach of menopause is signaled by a transitional period that begins with a break in the regular pattern of ovarian cycling. During the transitional phase, which lasts approximately 4 years, hormone levels fluctuate widely. In particular, FSH levels sometimes increase significantly, even in the presence of normal ovarian function and without an accompanying decrease in estradiol levels (Metcalf et al. 1981). Although overall levels of estradiol and inhibin change relatively little during the transitional phase (Korenman et al. 1978; Burger 1996*b*), subtle declines in their levels may contribute to the progressive elevation of FSH to consistently high postmenopausal levels (see next section of this article) (Burger 1996*b*).

## Hormones in Postmenopausal Women

Despite the absence of ovarian follicles, estrogen production continues after menopause, albeit at reduced levels. Postmenopausal estrogens are synthesized from a class of steroid hormones called androgens (e.g., testosterone and androstenedione). In men, androgens are the primary reproductive hormones, produced mainly in the testes. In women, androgens are produced in the ovaries and in the adrenal glands and are carried through the bloodstream to body fat, where androstenedione is converted to estrone (Korenman et al. 1978). After menopause, estrone replaces estradiol as the primary estrogen (Korenman et al. 1978). Postmenopausal estradiol levels are greatly reduced and are derived from the metabolism of estrone (Korenman et al. 1978; Burger 1996*a*).

Levels of testosterone and ovarian androstenedione also decrease after menopause, although adrenal androstenedione levels remain unchanged until later in life (Burger 1996*a*). Inhibin levels become undetectable after menopause (Burger 1996*b*). Released from the feedback regulation formerly provided by changing levels of ovarian hormones, levels of FSH increase up to 15 times compared with premenopausal levels, and LH levels increase up to 3 times (Burger 1996*b*); the significance of these increases in the absence of ovarian follicles is probably minimal.

## Alcohol and Estrogen Levels

Studies of alcohol’s effects on postmenopausal women generally focus on estradiol because of its greater potency compared with estrone. However, the low levels of estradiol after menopause are difficult to measure accurately (Carlstrom 1996). Methods for quantifying steroids differ in sensitivity and specificity, a factor that may contribute to the discrepancies in analytical results among studies (Gavaler et al. 1991; Cauley et al. 1989).

In one study, a single large dose of alcohol (42 to 46 grams, equivalent to approximately 3.5 to 4 standard drinks) caused a temporary increase in estradiol levels in postmenopausal women receiving HRT (Ginsburg et al. 1995, 1996) (see table 1), possibly by inhibiting the conversion of estradiol to estrone. However, alcohol administration had no effect on hormone levels in women who were not receiving HRT. This result is consistent with an earlier finding that alcohol administration under similar conditions had no effect on LH levels in postmenopausal women regardless of HRT use (Mendelson et al. 1985). In addition, alcohol administration had no effect on estrone levels in either group of women. The effects of lower doses or prolonged administration of alcohol have not been studied experimentally.

In nonexperimental studies, researchers have compiled epidemiological data to investigate possible associations between self-reported alcohol consumption and hormone levels among postmenopausal women (see table 2). Alcohol consumption was consistently unrelated to estrone levels in those studies. Findings for estradiol were mixed among women not on HRT, with some studies showing a direct association between alcohol consumption and estradiol levels and some not. In contrast to the results summarized in table 1, nonexperimental data on women receiving HRT (Johannes et al. 1997) found *lower* levels of estradiol among women who consume *higher* levels of alcohol (table 2).

Additional data suggest that alcohol consumption after menopause is unrelated to levels of androstenedione, (Cauley et al. 1989), testosterone (Cauley et al. 1989; Newcomb et al. 1995), or sex hormone-binding globulin, a specialized protein that facilitates the transport of androgens through the bloodstream (Newcomb et al. 1995; Nagata et al. 1997). However, two of three studies demonstrated an association between alcohol consumption and levels of a specific steroid substance that serves as an intermediary for the synthesis of androstenedione (Nagata et al. 1997; Barret-Conner and Goodman-Gruen 1995; Newcomb et al. 1995). The only study that examined alcohol’s effects on estrone sulfate found a positive association between alcohol consumption and estrone sulfate levels (Hankinson et al. 1995). Together, these data suggest that alcohol may affect hormones other than the major estrogens. However, confirmations of those associations are needed before any firm conclusions can be drawn.

The epidemiological studies described above address only the overall association between alcohol consumption and hormone levels. Little information is available on specific factors that might modify or contribute to alcohol’s effects. Those factors might include drinking patterns, including timing, frequency, and quantity of consumption; the time that has elapsed between a subject’s last drink and the determination of her hormone levels; and alcohol’s ultimate effects on the tissue responses induced by specific hormones. Additional data also are needed on the effects of alcohol on the adrenal steroid hormone precursors of estrogens and on estrone sulfate. In addition, most of the subjects in the studies cited who consumed alcohol were light or moderate drinkers (i.e., consuming approximately two to four drinks per week); thus, the available data provide little information about the effect of heavy drinking. Results also are subject to errors in the estimation of alcohol consumption and imprecision in the determination of hormone levels.

Overall, the data in tables 1 and 2 are consistent with the hypothesis that light or moderate alcohol consumption does not affect estrone levels. In addition, although data from some nonexperimental studies have linked alcohol consumption with higher levels of other steroid hormones in postmenopausal women (e.g., estradiol and estrone sulfate), the evidence that alcohol affects the levels of those hormones is inconclusive.

## Alcohol, Hormones, and Cancer

Cancer of the breast is among the most common fatal cancers among women in the United States. Annually, approximately 180,000 women are diagnosed with breast cancer, and 44,000 women die of the disease (Chu et al. 1996). Evidence suggests that alcohol consumption may be associated with increased risk for this cancer, although whether this association is causal remains unresolved (Longnecker 1992). The lack of strong evidence for alcohol-induced changes in estrogen levels either before or after menopause suggests that effects of alcohol in breast cancer may involve nonhormonal mechanisms. This conclusion is supported by evidence suggesting that alcohol consumption is not a risk factor for cancer of the glandular internal lining of the uterus (i.e., endometrial cancer), which is known to be hormone related (Rich-Edwards and Hennekens 1996). In the mammary glands of rats, alcohol caused tissue changes thought to be associated with increased risk for cancer without affecting estrogen levels (Singletary 1997).

## Alcohol, Hormones, and Heart Disease

Among women, consumption of at least one standard drink per day is associated with approximately a 20-percent reduction in risk of cardiovascular disease compared with nondrinkers (Thun et al. 1997). Although the nature of this relationship is the subject of continuing debate (Lowenfels 1998), evidence shows that moderate alcohol consumption ameliorates known risk factors for cardiovascular disease, including specific measures of blood chemistry and the clotting response (Freedman et al. 1992; Kannel and Ellison 1996). Estrogens have a beneficial effect on risk for cardiovascular disease, and most cardiovascular disease in women occurs after menopause, when estrogen levels are low (Witteman et al. 1998). Lack of a clear effect of alcohol on estrogens in postmenopausal women, however, does not preclude a causal relation between light and moderate intake and decreased risk of cardiovascular disease. (See the article by Gavaler on pp. 220–227 for another view on this topic.)

## Summary

The complete loss of ovarian follicles among middle-aged women is normal, and after menopause, estrogen levels are low. In general, the few studies available provide no strong evidence for an effect of alcohol on hormones in postmenopausal women. In addition, the possible association between alcohol consumption and risk of breast cancer does not appear to be mediated by estrogens. Evidence supports an association between moderate alcohol consumption and a decreased risk of cardiovascular disease, and this effect may be independent of hormonal influence. More data are needed to confirm these findings and to clarify the roles and relative importance of alcohol both during and after menopause on hormones other than estrogens.

## Figures and Tables

**Table 1:** Effect of a Single Dose of Alcohol on Estrogen Levels in the Blood of Postmenopausal Women

Author	*n*	Ethanol Dose (g)	Result		Subjects on Hormone Replacement Therapy

			E_1_	E_2_	
Ginsburg et al. 1995	7	46	no change	increased	yes
Ginsburg et al. 1996	12	42	no change	increased	yes
	12	42	no change	no change	no

NOTE: *n* = number of subjects; g = grams; E_1_ = estrone; E_2_ = estradiol.

**Table 2:** The Relation of Alcohol Consumption to Estrogen Levels in the Blood of Postmenopausal Women

Author	*n*	Result		Subjects on Hormone Replacement Therapy
		E_1_	E_2_	
Cauley et al. 1989	176^1^	no change	no change	past or never
Newcomb et al. 1995	253	no change	—	never
Hankinson et al. 1995	217	no change	no change	past or never
Johannes et al. 1997	148	no change	no change	past
	53	no change	no change	never
	92	no change	decreased	yes
Gavaler et al. 1991	244	—	increased^2^	past or never
Nagata et al. 1997	61	—	increased	past or never

NOTE: *n* = number of subjects; E_1_ = estrone; E_2_ = estradiol; — = not measured or data not given.

1 Note that 128 of the subjects were in the Pittsburgh subgroup of Gavaler et al. 1991.

2 E_2_ increased in subjects from three of four cities studied.

## Footnotes

1 The article cited defines menopause as the absence of menstrual periods for 1 year (Cramer and Xu 1996).

2 Although definitions of moderate drinking vary widely, an upper limit of two standard drinks per day for men and one for women is often employed. A standard drink is equivalent to 12 ounces of beer, 5 ounces of wine, or 1.5 ounces of 80-proof spirits (NIAAA 1992).

3 Unless otherwise specified, this article uses the term “level(s)” to refer to the concentrations of a given substance in the blood.

4 Osteoporosis is a bone disorder that commonly occurs in postmenopausal women (see article by Sampson and colleagues, pp. 190–194).
